# Expert Financial Advice Neurobiologically “Offloads” Financial Decision-Making under Risk

**DOI:** 10.1371/journal.pone.0004957

**Published:** 2009-03-24

**Authors:** Jan B. Engelmann, C. Monica Capra, Charles Noussair, Gregory S. Berns

**Affiliations:** 1 Department of Psychiatry & Behavioral Sciences, Emory University School of Medicine, Atlanta, Georgia, United States of America; 2 Department of Economics, Emory University, Atlanta, Georgia, United States of America; 3 Department of Economics, Tilburg University, Tilburg, The Netherlands; 4 Center for Neuropolicy, Emory University, Atlanta, Georgia, United States of America; Catholic University of Sacro Cuore, Italy

## Abstract

**Background:**

Financial advice from experts is commonly sought during times of uncertainty. While the field of neuroeconomics has made considerable progress in understanding the neurobiological basis of risky decision-making, the neural mechanisms through which external information, such as advice, is integrated during decision-making are poorly understood. In the current experiment, we investigated the neurobiological basis of the influence of expert advice on financial decisions under risk.

**Methodology/Principal Findings:**

While undergoing fMRI scanning, participants made a series of financial choices between a certain payment and a lottery. Choices were made in two conditions: 1) advice from a financial expert about which choice to make was displayed (MES condition); and 2) no advice was displayed (NOM condition). Behavioral results showed a significant effect of expert advice. Specifically, probability weighting functions changed in the direction of the expert's advice. This was paralleled by neural activation patterns. Brain activations showing significant correlations with valuation (parametric modulation by value of lottery/sure win) were obtained in the absence of the expert's advice (NOM) in intraparietal sulcus, posterior cingulate cortex, cuneus, precuneus, inferior frontal gyrus and middle temporal gyrus. Notably, no significant correlations with value were obtained in the presence of advice (MES). These findings were corroborated by region of interest analyses. Neural equivalents of probability weighting functions showed significant flattening in the MES compared to the NOM condition in regions associated with probability weighting, including anterior cingulate cortex, dorsolateral PFC, thalamus, medial occipital gyrus and anterior insula. Finally, during the MES condition, significant activations in temporoparietal junction and medial PFC were obtained.

**Conclusions/Significance:**

These results support the hypothesis that one effect of expert advice is to “offload” the calculation of value of decision options from the individual's brain.

## Introduction

**Figure 1:**
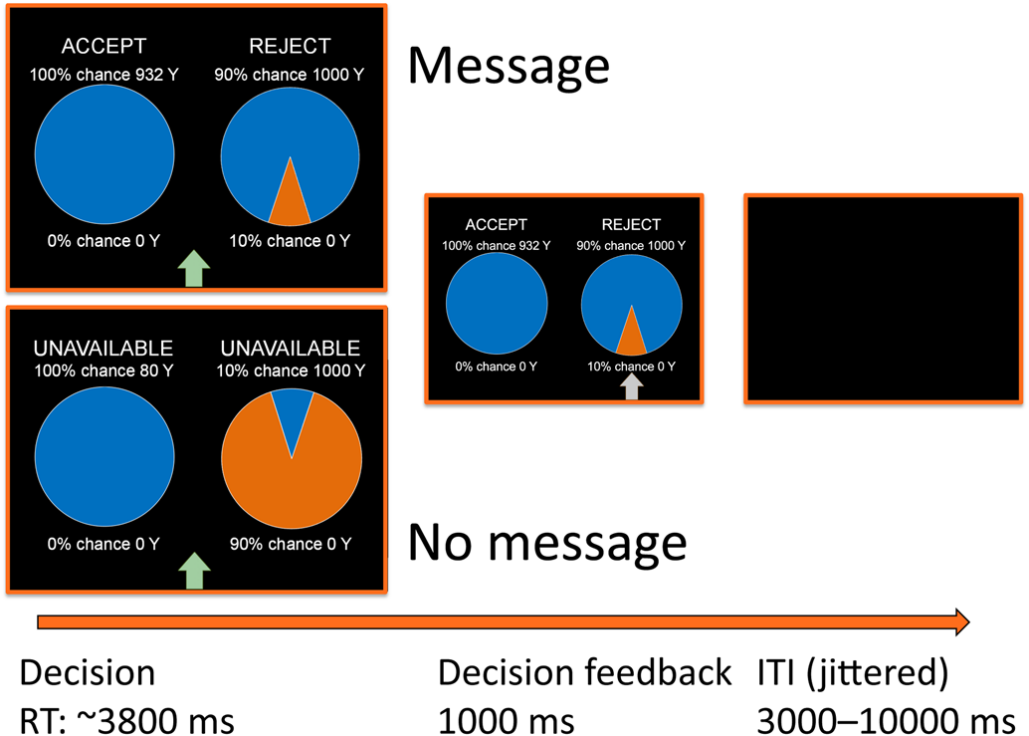
Schematic representation and timing of MRI trial design. On each trial, participants were asked to choose between a sure win and a lottery, either in the presence of advice from an expert (MESSAGE) or in its absence (NO MESSAGE). Advice from the expert economist was provided on half the trials by way of placing the words “ACCEPT” above the option that the expert would choose and “REJECT” above the option that the expert would not choose. In the NO MESSAGE condition, the expert's advice was hidden by placing the words “UNAVAILABLE” above both options. The probability of the lottery varied across seven probability conditions ranging from 1% to 99% and the amount of the sure win varied based on decision weights estimated in a behavioral pre-scanning session using the PEST procedure. The self-paced decision period was followed by a 1-second feedback period, which provided confirmatory information about which option was chosen by the participant. Finally, a jittered intertrial interval that varied between 3 and 10 seconds was presented.

Seeking advice from experts is common practice. The most prominent situations in which people turn to experts for advice occur under conditions of enhanced uncertainty, such as an economic recession. During such times, people may feel unfit to predict the consequences of their choices, and may seek the counsel of experts to reduce the enhanced perception of risk. For instance, when making investment decisions in a market downturn, people often ask an expert, or a knowledgeable colleague or friend for advice on where to invest their money. While the field of neuroeconomics has made significant progress in understanding the neurobiological basis of risk in decision-making (for reviews see [1], [2]), the neural impact of external information on decision-making, such as advice from an expert, remains unexplored. In the current experiment, we used functional magnetic resonance imaging (fMRI) to investigate the neurobiological basis of expert advice in a setting in which participants made financial decisions under uncertainty and were free to choose whether to follow or to ignore advice from a financial expert. Individuals made a series of financial choices between a certain payment and a lottery, while undergoing fMRI scanning. Choices were made under two different conditions. In one condition, advice from a financial expert was displayed, while in the second condition, no advice was displayed (Figure 1). The advice consisted of a recommendation from an expert economist about which choice to make. Our framing attempted to maximize the authoritativeness of the advice, with the purpose of creating a high likelihood that the advice would influence decisions.

There are several possible mechanisms through which expert advice could affect an individual's decision-making process [3], [4]. In particular, the impact of such advice may range along a continuum from having no effect on internal decision-making mechanisms to overriding them entirely. We hypothesized that advice would lead to modulations of internal valuation mechanisms engaged during choice, the effect of which we expected to be apparent both behaviorally and neurally. In the extreme, such modulations might take the form of suppressing, or turning off an individual's valuation mechanisms entirely. This would indicate an *offloading* of the burden of the decision-process to the expert. To test this *offloading* hypothesis, and to investigate the nature of the offloading process, we considered the following issues. 1) Does expert advice influence individuals' decisions? 2) What is the pattern of brain activations associated with the receipt of expert advice? 3) Is there a difference in brain activations between times when individuals choose to follow the expert's advice and when they do not? And 4) Does receipt of the message change activation patterns in brain regions associated with valuation computation when a decision is made?

To investigate the behavioral effect of expert advice (question 1), we compared decisions made during trials in which advice was received (which we refer to as the MES condition) and not received (the NOM condition). We used prospect theory [5] as a framework for analyzing how choices were affected by the presence of financial advice from an expert. Under this framework, the value of the lottery is the multiplicative product of the utility of each potential payoff and its subjectively weighted probability, both of which are susceptible to transformations. In particular, evidence suggests that subjects tend to overweight small probabilities and underweight large probabilities of an event [6], [7], [8], [9]. In the current experiment, we investigated whether and how probability weighting is affected by the expert's advice.

The second question was addressed by comparing brain activation patterns observed between the two conditions, MES (advice present) and NOM (advice absent). While information about the expert's professional background and his decision strategy were provided prior to the experiment in order to create a basic level of trust towards the expert, the quality of such information is not comparable to actual interactions with the expert. The specific nature of the expert's advice, which was suboptimal, could only be inferred via repeated interactions throughout the experiment. This process requires mental perspective-taking to infer the intentions and beliefs of the expert. Previous research in the field of social cognitive neuroscience has repeatedly associated such mental prespective-taking with activations in temporoparietal junction (TPJ) and medial prefrontal cortex (MPFC) (e.g. [10], [11], [12], [13], [14]). We therefore hypothesized that these regions (TPJ, MPFC) would be engaged when participants considered advice from an expert.

The third question was addressed by investigating activations when participants followed, compared to when they ignored, advice provided by the expert economist. Ignoring the advice of an expert may lead to enhanced conflict during the decision process and increase the perceived risk associated with choice. On the other hand, following the expert's advice can be considered a much safer option involving less emotional and cognitive conflict. We therefore expected non-conformity with the expert's advice to lead to enhanced levels of conflict and arousal. To investigate the neurobiological basis of this effect, we probed for differential activation patterns as a function of whether participants followed vs. ignored advice. Based on previous research [15], we expected brain regions associated with negative affect and risk, such as the anterior insula and amygdala, to show such enhanced activation when participants went against the expert's advice.

Finally, to investigate the neurobiological basis of the influence of expert advice during risky decision-making, we focused on activations in regions showing correlations with valuation and probability computations. In agreement with the *offloading* hypothesis, we expected signals reflective of value computations to be attenuated in the presence of expert advice. According to prospect theory, value computations reflect the multiplicative product of the utility of each potential payoff and its subjectively weighted probability. We expected the presence of expert advice to modulate the neural correlates of such value computations. This would be reflected by a reduced sensitivity to reward and probability magnitudes in regions previously associated with reward-related computations, such as the mesolimbic and mesocortical dopamine pathways [16], [17], [18], [19], [20], [21], and in regions implicated in probability processing, such as anterior and posterior cingulate cortex, anterior insula, parietal cortex and orbitofrontal cortex [22], [23], [24], [25], [26].

## Results

### Behavior

**Figure 2:**
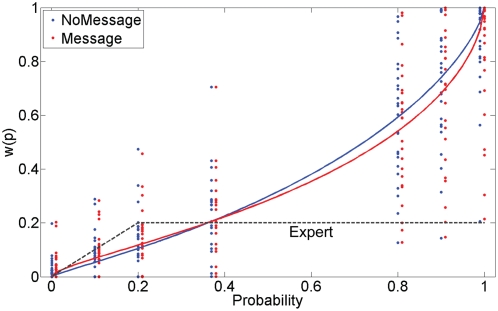
Probability weighting functions estimated from decisions in the MESSAGE and NO MESSAGE conditions. This figure shows probability weighting functions, *w*(*p*), and individual participants' decision weights at each probability condition estimated using our behavioral model. A significant difference in probability weighting functions between the NO MESSAGE (blue line) and MESSAGE (red line) condition was obtained. The differences indicate that participants overweighted probabilities smaller than 20% and underweighted probabilities greater than 80% to a greater extent in the MES compared to the NOM condition. These results indicate that the presence of the expert's advice, whose decision strategy is shown in the dotted line, had a significant effect on participants' probability weighting functions. A median α of 0.74 was obtained in the NO MESSAGE condition and, importantly, the α in the MESSAGE condition was significantly different, as indicated by a significant δ of −0.1066 (*P*<0.05).

Our behavioral results indicated that the expert's advice significantly influenced behavior. To investigate the extent to which expert advice affected individuals' estimated probability weighting parameters, we employed nonlinear logistic regression in combination with Prelec's compound invariant form [8], a commonly-employed specification for nonlinear probability weighting. Group-level parameter estimates agreed well with findings from behavioral economics (median α = 0.74; β = 1.59) and were consistent with an inverted S-shape probability function. We obtained behavioral evidence demonstrating that the presence of the expert's advice led to a significant increase in the curvature of w(p) in the direction of the advice, such that participants overweighted low probabilities and underweighted high probabilities more after receiving the advice. This was indicated by a significant estimate of δ (δ = −0.1066; 95% Confidence interval: [−0.1592 −0.054], see Figure 2). A complimentary method, using Certainty Equivalents obtained from the staircase algorithm employed in phase 2, yielded similar results (median α = 0.8964; β = 1.561; δ = −0.0442 [−0.1186 0.0302]). These findings demonstrate that the presence of the expert's advice led to a significant change in the curvature of the probability weighting function in the direction of the expert's advice. Reaction time results and proportion of trials during which expert advice was followed and ignored are shown in Table 1.

**Table 1:** Behavioral Results: Percentage of Trials in which Advice was Followed and Ignored in each Treatment Condition with associated Reaction Times, Average over All Subjects.

		Followed		Ignored	
		Percentage	RT	Percentage	RT
MES	Mean	72.81	3.169	27.19	4.561
	Std Error	4.07	0.244	3.45	0.883
NOM	Mean	64.10	3.231	35.90	4.092
	Std Error	4.07	0.226	3.45	0.468

### fMRI Results

#### Main effect of message

**Figure 3:**
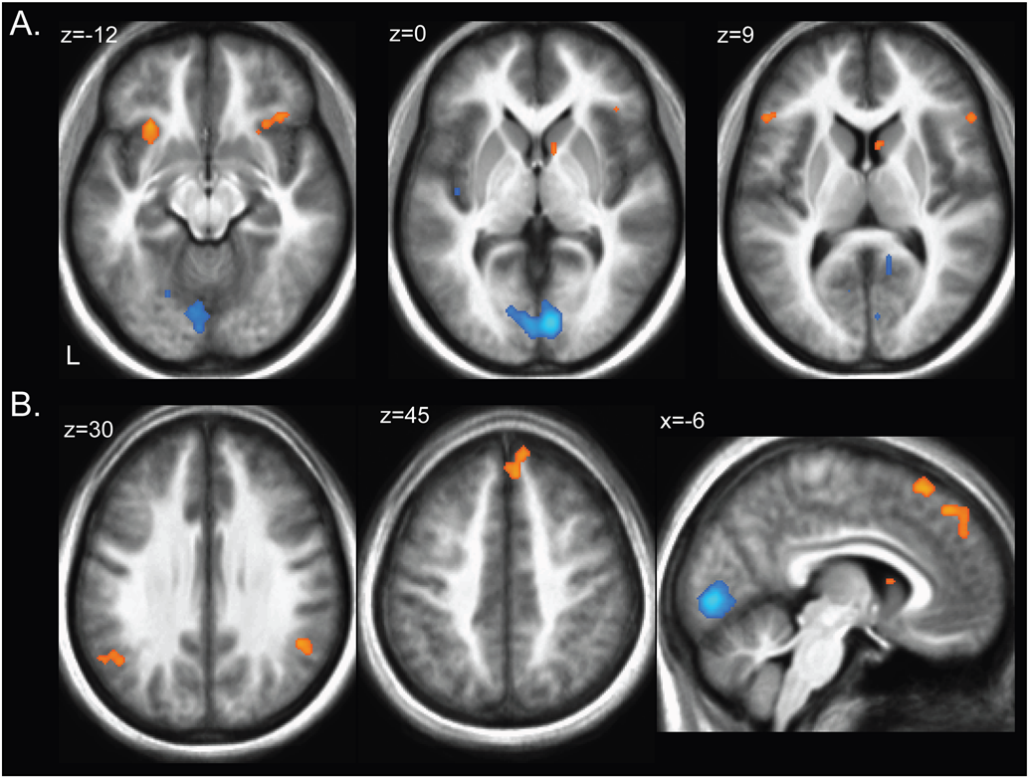
Activation clusters for the contrast *MESSAGE – NO MESSAGE* (p<0.001). Brain regions showing significant activations during the MESSAGE (*orange*) included bilateral anterior insula, right caudate nucleus, bilateral inferior frontal gyrus, bilateral temporoparietal junction and dorsomedial prefrontal cortex. Brain regions showing significant activations during the NO MESSAGE condition (*blue*) included lingual gyrus, fusiformgyrus, and left posterior insula.

To isolate areas activated during the message condition, we investigated the main effect of the message, contrasting presence and absence of the expert's advice (MES-NOM). As shown in Figure 3, structures showing significant activations during the message condition included bilateral temporoparietal junction (TPJ) and dorsomedial prefrontal cortex (DMPFC), as well as the caudate nucleus and anterior insula (see Table 2). Significant deactivations were reflective of greater responses in the NOM condition and were mainly observed in visual areas, as well as mid cingulate cortex and posterior insula (see Table 2).

**Table 2:** Brain Regions Showing Main Effect of Message.

L/R	Structure	BA	Volume	RL	AP	IS	Max t
*MESSAGE>NO MESSAGE*							
R	Superior Frontal Gyrus / DMPFC	8	101	3.5	38.4	51.2	6.654
L	SupramarginalGyrus / TPJ	40	37	−51.7	−57	31.2	4.564
R	SupramarginalGyrus / TPJ	40	31	50.9	−50.2	31.6	5.43
L	Insula	47	29	−29.4	18.2	−11.9	6.894
R	Insula	47	24	35.7	25	−11.3	4.8
R	Inferior Frontal Gyrus	47	8	55.5	25.5	7.5	4.028
R	Caudate		6	7.9	9.5	11.4	4.456
R	Caudate		5	9	8.4	1.2	4.552
L	Inferior Frontal Gyrus	47	5	−51.6	25.8	9	4.037
*NO MESSAGE>MESSAGE*							
R	Lingual Gyrus	18	263	1.2	−81.6	−3.2	−10.558
L	Cuneus	18	9	−9	−66	4	−4.266
R	Mid Cingulate Cortex	32	9	10.6	6.7	36.3	−4.952
L	FusiformGyrus	19	7	−19.7	−69.9	−9.4	−4.428
R	Posterior Cingulate Cortex	30	7	12.4	−50.9	10.3	−4.31
R	Middle Temporal Gyrus	22	6	58.5	−35.5	−3.5	4.421
L	Posterior Insula	22	5	−41.4	−13.8	−1.8	−4.402

#### Main effect of conformity

**Figure 4:**
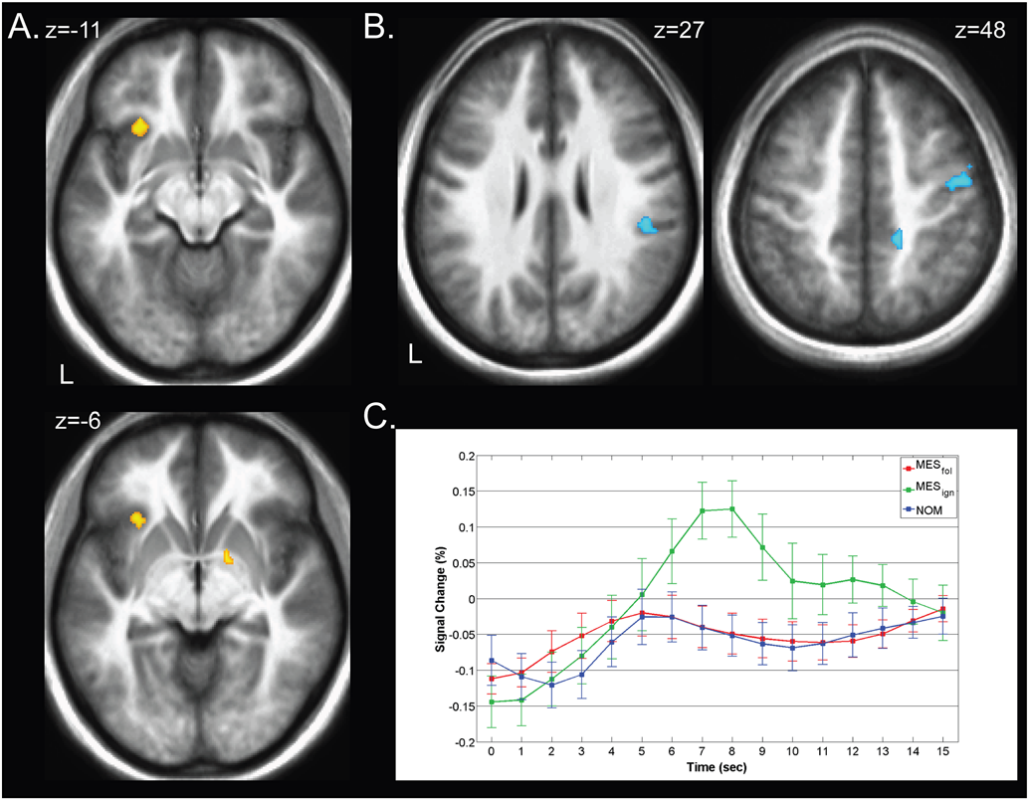
Neural correlates of conformity were revealed in the message condition by contrasting participants' choices to follow vs. ignore the advice of the expert (MES_fol_-MES_ign_). A. Brain regions responding when participants ignored the expert's advice included left anterior insula and right globus pallidus. B. Brain regions showing significant activation when participants conformed to the expert's advice included posterior insula, frontal eye fields and posterior cingulate, as well as two activation clusters in superior frontal gyrus (not shown here). C. The time course in the anterior insula shows a significant increase in activation when participants decided to ignore the expert's advice (*green line*) compared to when they decided to follow it (*red line*), or during the absence of the expert's advice in the NOM condition (*blue line*).

To probe for the effects of conformity, we contrasted blood oxygenation-level-dependent (BOLD) responses during trials in which participants chose to ignore versus follow the expert's advice in the message condition only. As shown in figure 4A, significant activations were obtained in the left anterior insula and right globus pallidus, indicating significant BOLD responses in these regions when subjects made decisions independently and ignored the expert's advice (see Table 3). A network of structures showed activations associated with conformity with the expert and included posterior cingulate, frontal eye fields, superior frontal gyrus, and posterior insula, all of which were lateralized to the right hemisphere (see Figure 4B and Table 3). Using a separate model to extract time courses in these regions, we confirmed that activity in regions associated with independence increased when subjects ignored the advice of the expert (MES_ignored_) compared to both when they followed it (MES_followed_) and when no advice was present (NOM), while the opposite pattern was observed in areas associated with behavioral conformity. Figure 4C shows the time course in the left anterior insula.

**Table 3:** Brain Regions Showing Effect of Ignoring vs. Following the Expert's Advice in the Message Condition.

L/R	Structure	BA	Volume	RL	AP	IS	Max t
*FOLLOWED>IGNORED*							
R	Posterior Insula	40	15	49.8	−31.5	28.4	−4.889
R	PrecentralGyrus / FEF	4	18	49.2	−7.4	48.7	−4.9174
R	Posterior Insula	5	15	17.6	−35.1	50.3	−5.3276
R	Superior Frontal Gyrus	6	5	8.4	−7.8	60	−4.3113
R	Superior Frontal Gyrus	6	6	14.5	−2	69.5	−4.0575
*IGNORED>FOLLOWED*							
L	Anterior Insula	47	21	−30.3	20.5	−9	4.9924
R	Globus Pallidus		6	15.5	2	−5.1	4.9392

#### Correlations with certain payoff and weighted value of lotteries

**Figure 5:**
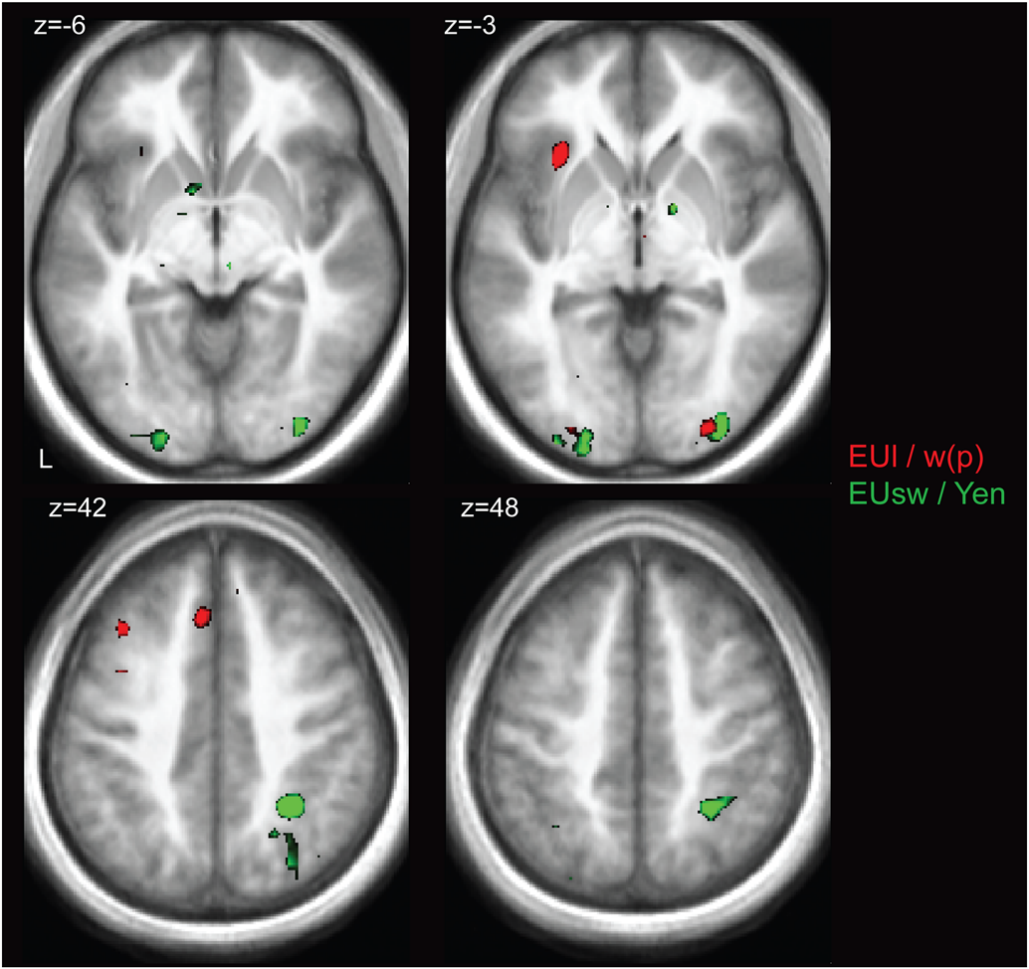
Valuation networks. Brain regions sensitive to weighted value of either the lottery (*red* probability weighting), or value of the sure win (*green*, magnitude) (P<0.001). Magnitude sensitive regions were determined using a linear contrast of sure win magnitudes offered to individual subjects. Regions sensitive to weighted lottery value were identified using a linear contrast of probability weights estimated for each individual subject, using our behavioral decision-making model. The networks for probability weighting and sure win magnitude were largely separate, except for overlap in occipital cortex, with parietal regions (intraparietal sulcus, precuneus), occipital cortex, as well as striatum being responsive to magnitude. Regions associated with nonlinear probability weighting included the anterior insula, dorsolateral PFC, anterior cingulate cortex and occipital cortex.

We probed for brain regions showing correlations with the weighted value of the lotteries (1000*w(*p*)). Because the payoff in the event of a win was always the same, these correlations are with w(p). Separately, we searched for brain regions with activation correlated with the magnitude of the sure win (recall that the amount of the sure win varied by trial). The MES and NOM conditions were investigated separately. Of note, parametric modulators for *w*(*p*) were orthogonalized to regressors reflective of sure win magnitude. In the MES condition, no significant correlations with the weighted value of the lottery were obtained, while cuneus and middle frontal gyrus showed negative correlations (deactivations) with the value of the sure win (see Table 4). In the NOM condition, we found significant correlations with the sure win in a network of structures consisting of intraparietal sulcus, posterior cingulate cortex, cuneus and precuneus, inferior frontal gyrus and middle temporal gyrus (see Figure 5 and Table 4). Significant correlations with the weighted value of the lottery were obtained in left anterior insula, anterior cingulate cortex, thalamus, precentral gyrus, and middle occipital gyrus (see Figure 5 and Table 4).

**Table 4:** Brain Regions Showing Correlations with Weighted Value of Lotteries and Magnitude of Sure Win during the NOMESSAGE (NOM) and MESSAGE (MES) conditions.

L/R	Structure	BA	Volume	RL	AP	IS	Max t	NPRR_99_ NOM>MES
*NOMESSAGE Lottery Value*								
L	Anterior Insula	47	15	−31.4	20.8	−2.1	5.058	No
L	Medial Frontal Gyrus	8	14	−6.2	27.7	42.9	4.7863	Yes
L	Thalamus		9	−0.7	−13	0.6	4.3643	Yes
L	Anterior Cingulate Cortex	9	7	−37.7	23.6	41.2	4.6137	Yes
R	Middle Occipital Gyrus	18	6	28.1	−89.5	−3.5	4.7843	Yes
*NOMESSAGE Sure Win Magnitude*								
R	IntraparietalSulcus	7	44	31.3	−48.5	44.2	5.8676	Yes
L	Cuneus	18	35	−23.9	−95.4	−8.3	5.2682	Yes
R	Middle Occipital Gyrus	18	31	32	−92	−1.1	4.9522	Yes
R	Precuneus	19	26	29.7	−64.8	40.5	4.4483	Yes
L	Cuneus	30	21	−11.3	−73	6.4	4.4809	Yes
R	Posterior Cingulate Cortex	30	13	19.6	−68.8	4.4	4.3002	Yes
R	Middle Temporal Gyrus	37	5	59.5	−49.3	−12	4.998	Yes
L	Middle Occipital Gyrus	19	5	−27	−60.6	1.1	4.9919	Yes
R	Posterior Cingulate Cortex	30	5	28.8	−65.4	7.2	4.2667	Yes
R	Inferior Frontal Gyrus	9	5	40.8	7.2	30	3.9422	No
L	Caudate		4	−9.0	7.5	−6.0	4.2107	No
*MESSAGE Sure Win Magnitude*								
L	Cuneus	18	7	−27	−20.1	52.7	−4.204	Yes
L	Middle Frontal Gyrus	4	6	−58.4	−35.1	−5.5	−4.5856	Yes

**Figure 6:**
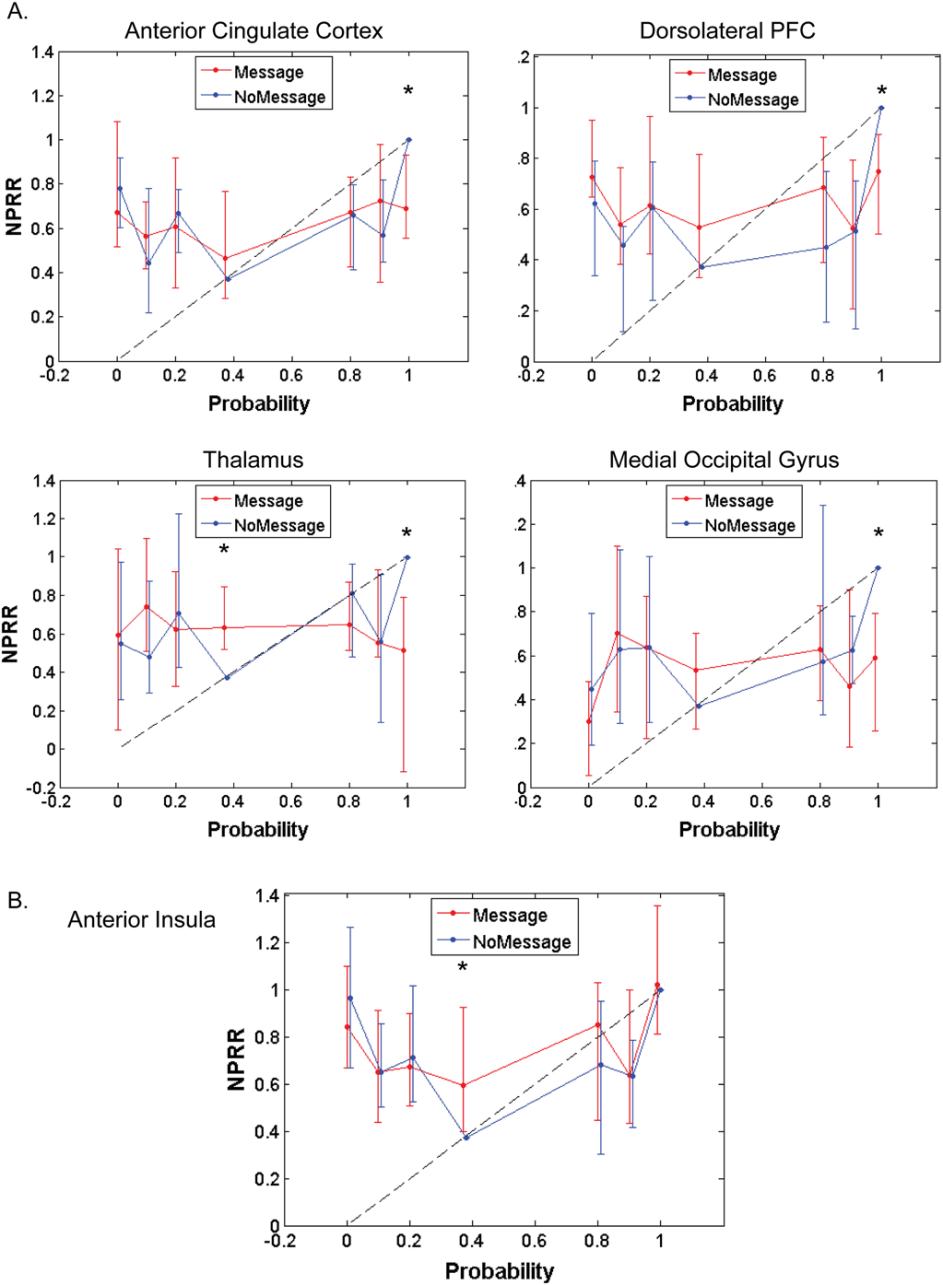
Neurobiological probability response ratios (NPRR) in representative regions showing correlations with weighted value of lottery (Table 4). The mean ratio for each subject was computed at each probability relative to the NOM baseline, and the median across subjects is plotted with error bars indicating the 95% confidence interval for the median. The NPRR curves are plotted with reference to the diagonal, which indicates linear probability weighting. Significant differences between NOM and MES function, denoted with “*”, were obtained consistently in the 99% probability condition (see Table 4), the point at which the effect of the expert's advice was maximal.

To consider whether the profile of the relationship between brain activation and probability differed between MES and NOM conditions in areas showing significant correlations with value, we obtained separate neurobiological probability response ratios (NPRRs) for the MES and NOM conditions. Because we did not observe any significant correlations with w(p) in the MES condition, we expected to find relatively flat NPRRs in the MES condition compared to the NOM condition. This would be reflected by significant differences between NPRRs in the two treatments, particularly for high probabilities, during which the expert advice was maximally different from risk neutrality or expected value maximization. Our findings are consistent with this hypothesis. Significantly greater responses in the NOM compared to the MES condition were obtained in the majority of areas showing correlations with w(p), or equivalently, with weighted value. This indicates extensive recruitment of valuation mechanisms in the absence of the expert's advice, and attenuation (offloading) when the expert's advice is present. Figure 6 shows representative NPRRs in regions showing significant correlations with probability weighting, including anterior cingulate cortex, dorsolateral PFC, thalamus, medial occipital gyrus and anterior insula.

## Discussion

A simple financial decision-making task involving risk was employed in the current study to investigate the behavioral and neural mechanisms by which financial advice, provided by an expert economist, affected decisions under risk. Behavioral results showed a significant effect of expert advice on probability weighting, such that probability weighting functions changed in the direction of the expert's advice. The behavioral effect of expert messages was paralleled by changes in neural activation patterns. Of note, significant correlations with the value of the lottery were obtained in the absence of the expert's advice (NOM), but not during its presence (MES). These results support the hypothesis that one effect of expert advice is to “offload” calculations of the value of alternative behavioral options that underlie decision-making from the individual's brain.

### Attenuated recruitment of valuation mechanisms parallels behavioral results

The significant behavioral effect of expert advice on probability weighting was paralleled by the fMRI results. At the behavioral level, we obtained a significant effect of the expert's advice on the curvature of the probability weighting function, indicated by a significant effect of the presence of the advice on α. Neurally, this was paralleled in two ways: 1) an attenuated activation of regions whose BOLD responses showed significant correlations with value in the NOM condition; and 2) significantly flattened NPRRs, reflecting an attenuated relationship between activation level and probabilities in regions that showed correlations with probability, in the MES condition relative to the NOM condition. Specifically, our results revealed that, in the absence of the expert's advice, participants engaged two largely separate networks involved in valuation mechanisms. These networks were composed of regions exhibiting correlations with two types of value, namely (a) payoff of the sure win and (b) weighted value of the lottery. These correlations were attenuated when the expert's advice was available.

These results implicate particular networks in evaluating the different behavioral options and underline their importance in the financial decision-making process. Regions that showed sensitivity to payoff magnitudes of prospects in the current study have previously been associated with decision-making under uncertainty, such as the parietal cortex, including precuneus [27] and intraparietal sulcus (IPS) [28], [29]. The IPS is the putative human homologue of LIP [30], an area in monkey parietal cortex that has previously been demonstrated to process elements of expected utility [28], [31] and has been proposed to contain a map of the expected utility of all possible actions [32]. In the current study, activity in parietal regions was associated with the value of the potential sure win. The posterior cingulate cortex/cuneus showed similar activation patterns, such that activity in this region was correlated with magnitude. These findings confirm and extend previous results implicating this area in decision-making under uncertainty [22], [33], [34], [35]. Specifically, responses in this area have been shown to scale with reward magnitude and predictability of reward [36] and PCC is an integrating node of the brain's default network involved in processing self-referential states (e.g. [37], [38]) and part of a network implicated in encoding *subjective* values of rewards [33], [34]. Importantly, the effect of the expert's advice was to attenuate these responses, which suggests that the advice, in part, “offloaded” the processing of value.

### The neurobiological equivalent to probability weighting is influenced by expert advice

We also confirm the presence of nonlinear weighting of probability, both at the behavioral, as well as the neural level in a network of areas sensitive to probability. We show that the degree of probability weighting can be influenced by the presence of advice from a financial expert, both at the behavioral (Figure 1) and, in parallel fashion, at the neural level (Figure 6). Neurally, the effect of the expert's advice was investigated using nonlinear probability weighting functions (NPRR, [26]). Specifically, we found that regions showing significant correlations with probability exhibited *flattened* NPRRs in the MES condition relative to the NOM condition. This effect was reflected in significantly lower NPRRs in the MES condition for probabilities above 80%.

In previous work, we demonstrated the presence of nonlinear probability weighting functions in a network of areas when probabilistic information about an impending electrical shock was provided [26]. Here, we extend our previous results by demonstrating (a) the presence of nonlinear probability weighting in the context of financial decision-making (that is, in which money is the reward medium) and, importantly, (b) that neurobiological probability response ratios are significantly biased by our experimental manipulation – advice from a “satisficing” expert economist. In the current study, areas showing significant correlations with nonlinear probability weighting included the anterior insula, anterior cingulate cortex, dorsolateral PFC, thalamus and medial occipital gyrus. Regions within this network also showed significantly flattened NPRRs in the presence of expert advice, especially at higher probabilities. These results confirm findings from previous research demonstrating nonlinear probability weighting in anterior cingulate cortex [23], [26]. Similarly, the anterior insula has repeatedly been implicated in risky decision-making and is thought to encode the negatively valenced affective aspects involved in risk [39], [40], [41], [42].

### Offloading to areas associated with mentalizing in the MESSAGE condition

Recruitment of valuation processes reflective of reward magnitude and probability weighting was greatly attenuated in the MES condition. Together with our behavioral results, these findings indicate that the presence of the expert's advice significantly altered the decision-making process. During the presence of the expert's advice, a network of brain regions was active that included areas associated with mentalizing others' intentions, such as TPJ and DMPFC (e.g. [10], [39], [43], [44]. Further regions showing increased activation during the presence of advice included the caudate nucleus, a region involved in social reward learning [45], [46], the anterior insula, a region involved in risk and negative emotional arousal ([47], [48]), and lateral inferior frontal gyrus (BA47), a region involved in inhibitory control and task switching ([49], [50]). Except for the anterior insula, these regions did not show overlap with areas involved in making financial choices. This activation pattern is consistent with the hypothesis that differential evaluatory processes were engaged when advice was provided by the expert compared to when participants made choices independent of the expert's advice.

Taken together, the activation pattern obtained in the presence of the expert's advice indicates an attenuated recruitment of valuation mechanisms that was accompanied by significant activations in regions associated with TOM reasoning. The TPJ, especially in the right hemisphere, has previously been associated with judgments of true and false beliefs that other people may hold [10], [51], as well as mental state attribution [10], [14] and belief attribution in the context of moral digressions [13]. The medial prefrontal cortex, on the other hand, is activated in a number of scenarios involving mentalizing [43], [52], [53], [54], [55], [56], [57]; for a review see [11]), with a region of dorsomedial prefrontal cortex being engaged when mentalizing about a dissimilar other [44]. These results parallel findings from the current study. Activation patterns in TPJ and DMPFC show increased BOLD responses in the presence of the expert's advice in a manner consistent with their involvement in belief attribution (TPJ) and reasoning about dissimilar others (DMPFC), which is a fitting description of the expert economist in relation to our student subject pool.

Activations in TOM areas during the presence of the expert's advice were accompanied by activations in regions associated with valuation in the context of decision-making tasks, including anterior insula and caudate nucleus. Previous research has implicated the caudate in feedback processes that guide future actions [58], [59]. Of particular importance to the current results are findings demonstrating that responses in the caudate are also involved in learning about the trustworthiness of trading partners in the context of economic games [45], [46]. Such studies have shown that responses in the caudate decreased as participants learned to make predictions about actions from partners in a manner consistent with a *social* reward-prediction error signal in this region [46]. Furthermore, responses in the caudate have been shown to be modulated by moral character descriptions when participants made risky financial choices about whether to trust trading partners in the context of a trust game [45]. In particular, the caudate responded to financial feedback about risky choices to trust a trading partner when little prior information about the partner's character was provided (neutral description), but not so when descriptions were informative about the trading partner's actions (good or bad descriptions). In view of the above findings, results from the current study are consistent with the hypothesis that the caudate nucleus is involved in social reward learning. It is possible that the caudate activations observed in the presence of the expert's advice in the current study may reflect learning signals about the trustworthiness of our expert.

### Nonconformity with the expert

To investigate the involvement of areas in conformity with, or independence of the expert, we probed for brain regions showing differential responses when following vs. ignoring the expert's advice. Regions showing increased responses when ignoring the expert included the anterior insula and globus pallidus, implicating these regions in nonconforming decisions that override the expert's advice. Interestingly, responses of a subset of globus pallidus neurons have recently been associated with negative reward-related signals [60], [61], which is consistent with the notion that activity in this region may reflect a negative evaluation of the expert's advice.

Little is known about the neurobiological basis of conformity. In a previous study, participants made binary perceptual decisions about rotated 3D objects in the presence of answers provided by either peers or a computer [15]. Conformity with the peer group was associated with increased activations in occipital and parietal regions, while independence of the peer group was associated with activations in right amygdala, an area associated with negative emotional states ([62]), and the head of the right caudate, activity of which is associated with stimulus salience ([63], [64], [65]). Overall, the findings indicate social modulation of perceptual regions during conformity and the presence of emotional salience and negative emotional arousal during independence.

These findings are corroborated by results from the current study demonstrating increased responses in the anterior insula when participants ignored the expert. The anterior insula has repeatedly been implicated in risky decision-making and is thought to encode the negatively valenced affective aspects involved in risk [39], [40], [41], [42], [43]. Here we demonstrate that activity in this area correlates with nonlinear probability weighting, as shown previously [26]. Additionally, our results implicate this area in evaluating the advice provided by the expert, as indicated by increased activity in this area during the presence of the expert's advice and when participant's decided to ignore the expert's message (Figure 4C). Thus, our findings suggest that the anterior insula is an integrative region that assesses the risk involved in choosing the lottery against the advice provided by the expert.

In summary, our results demonstrate that financial advice from an expert economist, provided during decision-making under conditions of uncertainty, had a significant impact on both behavior and brain responses. Behavioral results showed a significant effect of expert advice, such that probability weighting functions changed in the direction of the expert's advice. The behavioral effect of expert messages was paralleled by neural activation patterns. Specifically, (1) significant correlations with the value of choice alternatives were obtained only in the absence of the expert's advice, but not during its presence. This indicates an attenuation in the engagement of valuation processes in the presence of expert advice; (2) during the message condition, areas associated with mentalizing, such as DMPFC and bilateral TPJ were recruited, and finally, (3) ROI analyses of regions associated with probability indicated a significant “flattening” of neurobiological probability response ratios (NPRR) in the message condition compared to the no-message condition. This lends further support to the hypothesis that in the presence of advice from an expert, recruitment of valuation mechanisms was attenuated. Taken together, these results provide significant support for the hypothesis that one effect of expert advice is to “offload” the calculation of expected utility from the individual's brain.

## Methods

### Subjects

24 healthy, right-handed participants (15 females) participated in the current study, which was approved by the Emory University Institutional Review Board. The average age was 23 with a standard deviation of 5.3 years. The majority of participants were undergraduate students (17), 6 participants had graduate-level education, 1 subject chose not to provide educational information. All participants gave written informed consent and reported good health with no history of psychiatric disorders.

### Certainty Equivalent (CE) task

Each session of the experiment consisted of two phases. The first phase, was conducted outside the scanner and subjects made choices between a sure win and lotteries providing ex-ante probabilities of winning a comparatively higher payoff, as shown in Figure 1. The second phase proceeded in an identical fashion, except that individuals underwent scanning and advice from an expert economist was displayed on half the trials. The lotteries were specified so that the probability of winning the lottery varied across conditions (0.01, 0.1, 0.2, 0.37, 0.8, 0.9, 0.99). While the amount the individual received by winning the lottery was constant across trials (1000 units of “Yen,” the experimental currency), the value of the sure win was adjusted according to decisions made by the subject, as outlined in detail below.

In order to control for wealth effects, participants received a chance to win cash-rewards at the end of each session by randomly selecting one of the trials via a throw of three 10-sided dice. The decision made on the selected trial determined payment as follows: if the sure win was chosen on the selected trial, the respective amount was paid to the subject; if the lottery was chosen, a “computerized coin was tossed” giving subjects a chance to win 1000 laboratory Yen at the probability indicated in the lottery. An exchange rate of 1000 laboratory Yen = 16 USD was established at the beginning of the experiment, and subjects were informed of this rate. We used 1000 YEN as the lottery amount to facilitate the computation of the expected value of the lottery (should the subject wish to make such a calculation).

We optimized order and timing of our experimental design for adequate estimation of message- and probability-related responses [66], by randomly generating 5000 sequences of trials and choosing the best sequence based on a statistical efficiency criterion. Specifically, each experimental sequence included 224 trials in 7 Probability conditions (0.01, 0.1, 0.2, 0.37, 0.8, 0.9, 0.99), and occurring in the presence and absence of the expert's advice.

As shown in Figure 1, each trial included a 3.5-second decision-making period (based on the mean reaction time in pilot experiments), followed by a 1-second feedback period providing confirmatory information about which option was selected by the participant. Finally, an intertrial interval (ITI) that was drawn from a randomly generated exponential function with a mean of 5000 ms (range: 3000–10000 ms) was presented. At each iteration, trial order and ITI length were pseudo-randomized (no probability condition was repeated more than twice in a row, mean ITI length for all conditions was equalized) for all trials in a given experimental sequence and a design matrix was generated using AFNI's 3dDeconvolve script, assuming a TR of 2.5 seconds. The sequence with the smallest mean standard deviation for regressors of interest was chosen as the optimal sequence for use during scanning.

### Phase 1: Certainty Equivalent estimation using PEST

It has previously been argued that subjects can exhibit inconsistent preference behaviors, show strong framing effects, and distort reward magnitude or reward probability [67]. We therefore employed an iterative procedure during a behavioral pre-scanning session to obtain the best possible estimate of each participant's certainty equivalent (CE), or point of indifference between a lottery and a sure win. Having an estimate of an individual's CEs allowed us to optimize the range of offers presented during the scanning session. Specifically, to estimate each participant's CE, a modified version of the parameter estimation by sequential testing (PEST) procedure was employed [68], [69]. This staircase algorithm started with a random offer depending on probability condition, such that starting offers between 0 and 500 YEN were provided in low probability conditions (0.1–0.37) , while starting offers between 500 and 1000 YEN were provided in high probability conditions (0.8–0.99). In order to create choice switches between sure wins and lotteries, amounts for sure wins were adjusted as follows: whenever the subject chose the sure win, the amount offered on the next trial was decreased by step-size, ε; whenever the subject chose the lottery, the amount of the sure win offered on the next trial was increased by ε. The magnitude of ε was determined by the following 4 rules adapted from [68], [69]: (1) the initial step-size was set to 1/5 of the difference between the maximum and minimum possible payoffs (ε = 200 YEN); (2) at each choice switch ε was halved; (3) ε was doubled after three successive choices of the same item; (4) values were bounded at the maximum (1000 YEN) and the minimum payoffs (0 YEN). This was done within each probability condition, which were presented in random order, and separate counters kept track of choices within each probability condition. The staircase algorithm terminated when the threshold step-size for a given probability condition was reached. This threshold was set to 25 YEN for all conditions, except for 0.01, 0.37 and 0.99, for which the threshold was set to 12.5 YEN. Phase 1 was conducted outside the scanner.

There are both advantages and limitations of using the PEST procedure. One advantage is that we can generate CEs without having to opt for an auction such as the Becker-deGroot-Marschak (BDM) procedure, which would be difficult to implement in the scanner. On the other hand, one disadvantage is that it is possible, at least in principle, for the procedure to be manipulated by highly sophisticated subjects. However, we did not observe any evidence indicating that participants employed strategies to manipulate the staircase procedure in order to increase their chances of winning more money. Such a strategy would have led to a substantial overweighting of small probabilities relative to typical levels, which we did not find. Furthermore, during debriefing at the end of the study, participants indicated that they had not identified any way to engage in strategic behavior.

### Phase 2: Scanning Session

Inside the scanner, subjects made choices between lotteries and sure wins in a similar fashion as in the behavioral pre-scanning session, except that an expert economist provided his suggestions during half of the trials. In order to make the economist trustworthy, participants were informed of the economist's credentials and achievements, as well as his preferred decision strategy, in detailed instructions, which read as follows: “An expert Economist (Professor Charles Noussair of the Department of Economics at Emory University) is going to tell you his preferred decisions on half the trials. Professor Charles Noussair, Ph.D., earned Bachelor's degrees in Economics and Psychology from the University of Pennsylvania, and Master's and Doctorate Degrees from the California Institute of Technology. He has taught at Purdue and Emory Universities, and been a visiting professor in Australia, Japan, France, and the Netherlands. He has consulted for NASA, the Federal Reserve and the French ministry of Agriculture and has published numerous articles in high-impact peer-reviewed scientific journals.” None of the participants had been acquainted with Charles Noussair previously.

The expert's suggestions followed approximately a satisficing rule, which were, in part, consistent with those of a decision-maker trying to maximize his probability of winning at least 200YEN. Specifically, in trials in which the sure win was below 200YEN, the expert's advice was the option that maximized expected value. In trials in which the sure win was greater or equal to 200YEN, the expert advised acceptance of the sure win. Suggestions were displayed at the top of the screen via placing the word “ACCEPT” above the recommended option, and “REJECT” above the option not recommended (see Figure 1). In the other half of the trials, the word “UNAVAILABLE” was displayed above both options, to indicate that the economist's recommendations were not provided on that trial. Participants were instructed to pay attention to and consider the expert's recommendations, but to make choices based on which option they considered most attractive.

During phase 2, the sure win magnitudes were based on CEs estimated during phase 1, and differed for each subject. Specifically, sure win magnitudes were selected randomly from the interval [CE−(0.4 * CE), CE+(0.4 * (1000-CE))], except for eight of the subjects, for which sure win magnitudes were provided by CE±0.2 * CE and CE±0.4 * CE (the discretized range was changed to a random sampling of the interval to provide better coverage of the offer space).

To allow for potential changes in CE which might occur if participants followed the expert advice during the scanning session, an attenuated version of the staircase algorithm was employed during phase 2 as well. This was done to ensure an adequate number of offers both above and below the subject's CE, even if the CE changed during the course of the experiment. This staircase algorithm tracked extreme behaviors, such that CEs in a given probability condition were adjusted when subjects deviated from expected behavior. Specifically, CEs were decreased by ¼ of the difference between CE and sure win magnitude in a given probability condition when participants accepted a sure win that was lower than CE, while CEs were increased by ¼ of the difference between CE and sure win magnitude, when participants chose to reject a sure win that was larger than the CE of the current probability condition. This adaptive algorithm tracked changes in probability weighting during the scanning session, which (1) ensured that sure win magnitudes were based on current decision-making parameters by accounting for potential changes in probability weighting that might occur as a function of context change (such as scanner environment, presence of expert, experienced outcome after completion of PEST procedure) and (2) provided a method for estimating the effects of expert messages on probability weighting that is independent of our behavioral decision making model outlined below.

### Decision Making Model

Empirical evidence suggests that decisions under risk are typically consistent with the transformation of objective probability, *p*, by a function, *w*(*p*), which has an inverted S-shape [6], [7], [8], [9], with *w*(*p*)>*p* for *p*<p*, *w*(*p*)<*p* for *p*>p*, and p*≈0.37. Probability weighting has been integrated as a key factor in several theories of choice under risk, including prospect and cumulative prospect theory [5], [70], as well as rank dependent utility theory [71].

We employed nonlinear logistic regression to estimate each participant's probability weighting and utility functions from their binary decisions (lottery or sure-win) using a modified version of Prelec's compound invariant form [8] with additional parameters estimating the effect of the message on probability weighting. In each trial, the participant had a choice between a sure win (SW) and a lottery. According to Prospect Theory, the value of the lottery is given by *∑_i_w*(*p_i_*)*U*(*x_i_*). Let *Φ*, be the difference in utility between the lottery and the sure win in each trial. We take this difference as the main determinant of our behavioral decision-making model.:
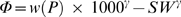
(1)where, on a given trial, *P* is the probability of winning the lottery, *SW* reflects the value of the sure win and γ reflects the curvature of the utility function. *w*(*P*) was modeled using a modified version of Prelec's compound invariant form, such that
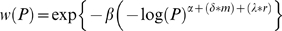
(2)where *m* is a dummy variable indicating the presence (1) or absence (0) of an expert message and *δ* captures the effect of message on *α*, *r* is run number and *λ* is a learning parameter. We will refer to *w*(*P*)×1000*^γ^* as the *Weighted Walue* of the lottery to an individual. The learning parameter was included to allow for the possibility that the expert advice affected decision-making across all trials, including non-message trials. The probability of choosing the lottery (*P_l_*) was estimated using a logistic regression specification:
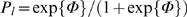
(3)where P_l_ is the probability of choosing the lottery. Parameters were estimated for each subject using the least squares curve fit function in Matlab. Starting parameters were obtained for *α*, *β*, *δ*, *λ*, and *γ* by iterating through a matrix of starting parameters varying between [−1 1.5] and choosing those starting parameters that minimized residuals for each subject. Finally, decision weights, *w*(*P*), were obtained for each subject separately. Decision weights were (a) used as parametric modulators in neuroimaging analyses and (b) entered into a random effects behavioral group-level model that used nonlinear regression to obtain decision weights as a function of the presence of the expert's advice using Prelec's compound invariant form with additional parameters for each participant's *α* and *δ*, where *δ* captures the influence of the message on α:
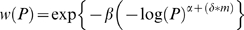
(4)


#### Functional Magnetic Resonance Imaging

Neuroimaging data were collected using a 3 Tesla Siemens Magnetron Trio whole body scanner (Siemens Medical Systems, Erlangen, Germany). A three dimensional, high-resolution anatomical data set was acquired using Siemens' magnetization prepared rapid acquisition gradient echo (MPRAGE) sequence (TR of 2300 ms, TE of 3.93 ms, TI of 1100 ms, 1 mm isotropic voxels and a 256 mm FOV). Functional data consisted of thirty-five axial slices that were sampled with a thickness of 3 mm and encompassing a field of view of 192 mm with an inplane resolution of 64×64 (T2* weighted, TR = 2500 ms, TE = 31 ms). The task was presented with Presentation software (Neurobehavioral Systems, Albany, CA) and visual stimuli were projected onto a frosted glass screen, which the subject viewed through an angled mirror mounted to the head coil. Inhomogeneities in the magnetic field introduced by the participant were minimized with a standard two-dimensional head shimming protocol before each run and the anatomical data acquisition. In our dataset, each participant completed 4 runs with 56 trials, whose length depended on participants' decision time.

### fMRI Data Analysis

#### fMRI Preprocessing

Initial preprocessing of the data was conducted using Analysis of Functional Neuroimages (AFNI, http://afni.nimh.nih.gov/afni). Data underwent slice-time acquisition correction using Fourier interpolation. The functional data were then spatially aligned to the volume acquired closest to each subject's anatomical image. After motion correction, anatomical and mean functional datasets were manually co-registered. Individual gray matter tissue probability maps (TPMs) were computed from anatomical datasets and spatially warped to standard MNI space using the VBM5 toolbox (http://dbm.neuro.uni-jena.de/vbm) running in SPM5 (http://fil.ion.ucl.ac.uk/spm/software/spm5). Normalization to standard MNI space was conducted in SPM5 by applying the transformation matrix obtained from normalizing the anatomical data set to the functional data using quintic interpolation. Functional data then underwent spatial smoothing using an isotropic Gaussian kernel (full width at half maximum (FWHM) = 6 mm). Finally, each voxel's signal intensity was scaled to a mean of 100.

#### fRMI Analysis

FMRI data were analyzed using the General Linear Model and a standard two-stage mixed effects analysis. Trials were classified according to type of decision made by participants. Specifically, responses were sorted according to whether participants *followed* or *ignored* the expert's advice in the message condition (MES), while in the no message condition (NOM) responses were sorted according to whether participants' would have followed or ignored the expert's advice had it been shown. The latter conditions were included in the analyses in order to control for potential learning effects across message conditions. First-level multiple regression models consisted of 4 regressors of interest, modeling the presence/absence of expert choice and type of decision made by the subject. Two additional regressors were included in each condition in the form of parametric modulators predictive of (1) amount of sure win magnitude (*SW*) offered on each trial, and (2) decision weights, *w*(*P*), reflective of subjective distortions of actual probabilities as estimated by the behavioral decision-making model outlined above. Of note, parametric modulators were tailored to each subject's level of risk attitude. Because we were interested mainly in the effects of expert messages on probability weighting, we orthogonalized the regressors reflecting parametric modulation by *w*(*P*) relative to regressors reflecting parametric modulation by *SW* within each condition. This procedure generated new regressors within each condition whose variance in *w*(*P*) is not explained by *SW*. All first-level statistical models included additional regressors of no interest for each run to model slow signal drifts (constant and polynomial terms), to account for residual head motion (roll, pitch, yaw, and displacement in superior, left and posterior directions) and one regressor to model the 1-second feedback period presented after each decision.

To localize regions involved in processing the presence of the expert (MES main effect), a random effects model was implemented at the second level as separate paired two-sample t-tests contrasting the beta images corresponding to 1) presence and the absence of the MES and 2) ignoring the expert and following the expert in the MES condition only. Finally, to probe for brain regions associated with valuation processes (probability weighting and magnitude of sure win), correlation contrasts were performed with separate one-sample t-tests on parametric modulators in MES and NOM conditions. All t-maps were thresholded at an uncorrected p-value of 0.001 with a cluster size threshold of k>5, except for striatal areas for which a cluster size threshold of k>4 was employed.

#### ROI analysis of regions showing conformity and independence with the expert

To extract the temporal dynamics within regions showing a significant response when subjects followed or ignored the expert's advice in the MES condition, a different first-level model was fitted to each participant's fMRI data. In this model, the hemodynamic response during MES and CHOICE was modeled with a basis set of seven cubic spline functions spaced one TR (2.5 s) apart and spanning the interval from 0 to 15 seconds post trial onset. In order to create reconstructed event-related responses on a 1 s temporal grid, the set of fitted spline functions was resampled at a temporal resolution of 1 second and averaged within each ROI as a function of the following condition of interest: MES_followed_, MES_ignored_ and NOM.

#### Neurobiological probability response ratio (NPRR)

The above whole-brain analysis probed for regions encoding non-linear probability weighting and sure win magnitude. To illustrate fMRI responses within structures associated with valuation, and, importantly, differences in probability weighting as a function of the expert's advice, we analyzed the ROI activations using a previously developed method of transforming neural activations to a neural analog of the probability weighting function. Specifically, we were interested in how the NPRR, the relationship between neural activation and probability of a given outcome, was affected by the presence of the expert's advice. This model was only used in areas already identified by the aforementioned contrasts. We converted mean activations reflecting signal change to neurobiological probability response ratios (NPRR) following methods described in detail in Berns et al. (2008). According to prospect tgheory, the probability weighting function, *w*(*p*), is a monotonic function that is bounded by [0,1] in both domain and range. The function transforms an objective probability, *p*, into a *weight* based on subjective factors when evaluating a lottery. An equivalent function based on fMRI responses can be obtained via transforming neural responses to the presentation of a probability, *y*(*p*). We assume that a null outcome, such as a payoff of zero, occurs with probability 1−*p*. The NPRR function is given by NPRR(*p*) = *y*(*p*)/*y*(1), where *y*(1) is the fMRI response to a lottery with *p* = 1. Because fMRI responses do not represent absolute physical quantities, a baseline response was defined as the BOLD response during the NOM condition at *p* = 0.37. This is approximately the point at which the probability weighting function crosses the identity function, as demonstrated by various behavioral studies [5], [6], [70], [72], [73]. This choice of baseline controls for various psychological and physiological processes associated with low-level processing of the stimuli [26]. The value of *y*(1), at outcomes which are certain, was approximated by the mean beta value of the BOLD response for those trials in the NOM condition in which the probability of winning the lottery was 0.99. The restrictions that *y_NOM_*(.99) = 1 and *y_NOM_* (.37) = .37 yield the following NPRR:
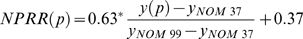
(5)NPRRs, were estimated separately for mean BOLD responses during both the message and no message conditions. As in [26], the median of the means were employed to estimate central tendency and a 95% confidence interval was obtained to estimate statistical significance using the following equation: [(N+1)/2]±1.96*(√N)/2 [74].

## References

[1] Platt ML, Huettel SA (2008). Risky business: the neuroeconomics of decision making under uncertainty.. Nat Neurosci.

[2] Schultz W, Preuschoff K, Camerer C, Hsu M, Fiorillo CD (2008). Explicit neural signals reflecting reward uncertainty.. Philos Trans R Soc Lond B Biol Sci.

[3] Deutsch M, Gerard HB (1955). A study of normative and informational social influences upon individual judgment.. Journal of Abnormal and Social Psychology.

[4] Bonaccio S, Dalal RS (2006). Advice taking and decision-making: An integrative literature review, and implications for the organizational sciences.. Organizational Behavior and Human Decision Processes.

[5] Kahneman D, Tversky A (1979). Prospect theory: An analysis of decision under risk.. Econometrica.

[6] Wu G, Gonzalez R (1996). Curvature of the probability weighting function.. Management Science.

[7] Gonzalez R, Wu G (1999). On hte Shape of the Probability Weighting Function.. Cognitive Psychology.

[8] Prelec D (1998). The probability weighting function.. Econometrica.

[9] Camerer C, Ho TH (1994). Violations of betweenness axiom and nonlinearity in probability.. Journal of Risk and Uncerstainty.

[10] Saxe R, Kanwisher N (2003). People thinking about people: the role of the temporo-parietal junction in “theory of mind”.. NeuroImage.

[11] Frith U, Frith CD (2003). Development and neurophysiology of mentalizing.. Philos Trans R Soc Lond B Biol Sci.

[12] Frith CD, Frith U (2006). The neural basis of mentalizing.. Neuron.

[13] Young L, Cushman F, Hauser M, Saxe R (2007). The neural basis of the interaction between theory of mind and moral judgment.. Proc Natl Acad Sci U S A.

[14] Saxe R, Wexler A (2005). Making sense of another mind: the role of the right temporo-parietal junction.. Neuropsychologia.

[15] Berns GS, Chappelow JC, Zink CF, Pagnoni G, Martin-Skurski ME (2005). Neurobiological correlates of social conformity and independence during mental rotation.. Biol Psychiatry.

[16] Schultz W (1997). Dopamine neurons and their role in reward mechanisms.. Current Opinion in Neurobiology.

[17] Mirenowicz J, Schultz W (1996). Preferential activation of midbrain dopamine neurons by appetitive rather than aversive stimuli.. Nature.

[18] Ljungberg T, Apicella P, Schultz W (1992). Responses of monkey dopamine neurons during learning of behavioral reactions.. Journal of Neurophysiology.

[19] O'Doherty JP, Deichmann R, Critchley HD, Dolan RJ (2002). Neural responses during anticipation of a primary taste reward.. Neuron.

[20] Elliott R, Friston KJ, Dolan RJ (2000). Dissociable neural responses in human reward systems.. Journal of Neuroscience.

[21] Ernst M, Nelson EE, McClure EB, Monk CS, Munson S (2004). Choice selection and reward anticipation: an fMRI study.. Neuropsychologia.

[22] McCoy AN, Platt ML (2005). Risk-sensitive neurons in macaque posterior cingulate cortex.. Nat Neurosci.

[23] Paulus MP, Frank LR (2006). Anterior cingulate activity modulates nonlinear decision weight function of uncertain prospects.. NeuroImage.

[24] Tobler PN, Fiorillo CD, Schultz W (2005). Adaptive coding of reward value by dopamine neurons.. Science.

[25] Fiorillo CD, Tobler PN, Schultz W (2003). Discrete coding of reward probability and uncertainty by dopamine neurons.. Science.

[26] Berns GS, Capra CM, Chappelow J, Moore S, Noussair C (2008). Nonlinear neurobiological probability weighting functions for aversive outcomes.. Neuroimage.

[27] Huettel SA (2006). Behavioral, but not reward, risk modulates activation of prefrontal, parietal, and insular cortices.. Cognitive, Affective, & Behavioral Neuroscience.

[28] Platt ML, Glimcher PW (1999). Neural correlates of decision variables in parietal cortex.. Nature.

[29] Shadlen MN, Newsome WT (2001). Neural basis of perceptual decision in the parietal cortex (area LIP) of the rhesus monkey.. Journal of Neurophysiology.

[30] Schluppeck D, Glimcher P, Heeger DJ (2005). Topographic organization for delayed saccades in human posterior parietal cortex.. J Neurophysiol.

[31] Sugrue LP, Corrado GS, Newsome WT (2004). Matching behavior and the representation of value in the parietal cortex.. Science.

[32] Glimcher PW, Dorris MC, Bayer HM (2005). Physiological utility theory and the neuroeconomics of choice.. Games and Economic Behavior.

[33] Small DM, Zatorre RJ, Jones-Gotman M (2001). Increased intensity perception of aversive taste following right anteromedial temporal lobe removal in humans.. Brain.

[34] Kable JW, Glimcher PW (2007). The neural correlates of subjective value during intertemporal choice.. Nat Neurosci.

[35] Vogt BA, Finch DM, Olson CR (1992). Functional heterogeneity in cingulate cortex: the anterior executive and posterior evaluative regions.. Cereb Cortex.

[36] McCoy AN, Crowley JC, Haghighian G, Dean HL, Platt ML (2003). Saccade reward signals in posterior cingulate cortex.. Neuron.

[37] Raichle ME, MacLeod AM, Snyder AZ, Powers WJ, Gusnard DA (2001). A default mode of brain function.. Proc Natl Acad Sci U S A.

[38] Buckner RL, Andrews-Hanna JR, Schacter DL (2008). The brain's default network: anatomy, function, and relevance to disease.. Ann N Y Acad Sci.

[39] Kuhnen CM, Knutson B (2005). The neural basis of financial risk taking.. Neuron.

[40] Huettel SA, Song AW, McCarthy G (2005). Decisions under uncertainty: probabilistic context influences activation of prefrontal and parietal cortices.. J Neurosci.

[41] Paulus MP, Rogalsky C, Simmons A, Feinstein JS, Stein MB (2003). Increased activation in the right insula during risk-taking decision making is related to harm avoidance and neuroticism.. Neuroimage.

[42] Sanfey AG, Rilling JK, Aronson JA, Nystrom LE, Cohen JD (2003). The neural basis of economic decision-making in the Ultimatum Game.. Science.

[43] Rilling JK, Sanfey AG, Aronson JA, Nystrom LE, Cohen JD (2004). The neural correlates of theory of mind within interpersonal interactions.. Neuroimage.

[44] Mitchell JP, Macrae CN, Banaji MR (2006). Dissociable medial prefrontal contributions to judgments of similar and dissimilar others.. Neuron.

[45] Delgado MR, Frank RH, Phelps EA (2005). Perceptions of moral character modulate the neural systems of reward during the trust game.. Nat Neurosci.

[46] King-Casas B, Tomlin D, Anen C, Camerer CF, Quartz SR (2005). Getting to know you: reputation and trust in a two-person economic exchange.. Science.

[47] Wicker B, Keysers C, Plailly J, Royet JP, Gallese V (2003). Both of us disgusted in My insula: the common neural basis of seeing and feeling disgust.. Neuron.

[48] Singer T, Seymour B, O'Doherty J, Kaube H, Dolan RJ (2004). Empathy for pain involves the affective but not sensory components of pain.. Science.

[49] Robbins TW (2007). Shifting and stopping: fronto-striatal substrates, neurochemical modulation and clinical implications.. Philos Trans R Soc Lond B Biol Sci.

[50] Derrfuss J, Brass M, Neumann J, von Cramon DY (2005). Involvement of the inferior frontal junction in cognitive control: meta-analyses of switching and Stroop studies.. Hum Brain Mapp.

[51] Grezes J, Frith CD, Passingham RE (2004). Inferring false beliefs from the actions of oneself and others: an fMRI study.. NeuroImage.

[52] Castelli F, Happe F, Frith U, Frith C (2000). Movement and mind: a functional imaging study of perception and interpretation of complex intentional movement patterns.. Neuroimage.

[53] Gallagher HL, Happe F, Brunswick N, Fletcher PC, Frith U (2000). Reading the mind in cartoons and stories: an fMRI study of ‘theory of mind’ in verbal and nonverbal tasks.. Neuropsychologia.

[54] Ferstl EC, von Cramon DY (2002). What does the frontomedian cortex contribute to language processing: coherence or theory of mind?. Neuroimage.

[55] McCabe K, Houser D, Ryan L, Smith V, Trouard T (2001). A functional imaging study of cooperation in two-person reciprocal exchange.. Proceedings of the National Academy of Science, USA.

[56] Schultz RT, Grelotti DJ, Klin A, Kleinman J, Van der Gaag C (2003). The role of the fusiform face area in social cognition: implications for the pathobiology of autism.. Philos Trans R Soc Lond B Biol Sci.

[57] Vogeley K, Bussfeld P, Newen A, Herrmann S, Happe F (2001). Mind reading: neural mechanisms of theory of mind and self-perspective.. Neuroimage.

[58] Tricomi EM, Delgado MR, Fiez JA (2004). Modulation of caudate activity by action contingency.. Neuron.

[59] O'Doherty J, Dayan P, Schultz J, Deichmann R, Friston K (2004). Dissociable roles of ventral and dorsal striatum in instrumental conditioning.. Science.

[60] Matsumoto M, Hikosaka O (2008). Negative motivational control of saccadic eye movement by the lateral habenula.. Prog Brain Res.

[61] Hikosaka O, Sesack SR, Lecourtier L, Shepard PD (2008). Habenula: crossroad between the basal ganglia and the limbic system.. J Neurosci.

[62] LeDoux JE (2000). Emotion circuits in the brain.. Annu Rev Neurosci.

[63] Zink CF, Pagnoni G, Martin-Skurski ME, Chappelow JC, Berns GS (2004). Human striatal responses to monetary reward depend on saliency.. Neuron.

[64] Zink CF, Pagnoni G, Martin ME, Dhamala M, Berns GS (2003). Human striatal response to salient nonrewarding stimuli.. Journal of Neuroscience.

[65] Zink CF, Pagnoni G, Chappelow J, Martin-Skurski M, Berns GS (2006). Human striatal activation reflects degree of stimulus saliency.. Neuroimage.

[66] Birn RM, Cox RW, Bandettini PA (2002). Detection versus estimation in event-related fMRI: choosing the optimal stimulus timing.. Neuroimage.

[67] Kühberger A, Schulte-Mecklenbeck M, Perner J (1999). The effects of framing, reflection, probability, and payoff on risk preference in choice.. Organizational Behavior and Human Decision Processes.

[68] Luce RD (2000). Utility of gains and losses: Measurement-theoretical, and Experimental Approaches: Lawrence Erlbaum.

[69] Cho Y, Luce RD, von Winterfeld D (1994). Tests of assumptions about the joint receipt of gambles in rank- and sign-dependent utility theory.. Journal of Experimental Psychology: Human Perception and Performance.

[70] Tversky A, Kahneman D (1992). Advances in prospect theory. Cumulative representation of uncertainty.. Journal of Risk and Uncertainty.

[71] Quiggin J (1993). Generalized Expected Utility Theory: The Rank-Dependent Model: Springer.

[72] Abdellaoui M (2000). Parameter-free elicitation of utility and probability weighting functions.. Management Science.

[73] Fehr-Duda H, de Gennaro M, Schubert R (2006). Gender, financial risk, and probability weights.. Theory and Decision.

[74] Woodruff RS (1952). Confidence intervals for medians and other position measures.. Journal of the American Statistical Association.

